# Can digital literacy promote farmers’ cultivated land quality protection behaviors?

**DOI:** 10.1371/journal.pone.0319050

**Published:** 2025-02-27

**Authors:** Shuang Hu, Huogen Wang

**Affiliations:** School of Economics and Management, Jiangxi Agricultural University, Nanchang, Jiangxi, China; Huaqiao University, CHINA

## Abstract

Cultivated land is the most valuable agricultural resource and the most important factor of production. The protection of cultivated land quality is not only the foundation of national food security but also a strategic issue related to the sustainable development of the economy and society as a whole. This paper uses survey data from Jiangxi Province’s “Double Hundred and Thousand” in 2023 and empirically analyzes the impact of digital literacy on farmers’ cultivated land quality protection behaviors and its mechanism by using the O-probit model and the mediating effect model. The results show that digital literacy can effectively promote farmers’ cultivated land quality protection behaviors. Furthermore, digital literacy can indirectly promote farmers’ cultivated land quality protection behaviors through cultivated land protection cognition and digital use ability. Heterogeneity analysis indicates that farmers are more inclined to participate in cultivated quality protection behaviors In the context of better soil fertility conditions and higher digital literacy. Therefore, it is necessary to continuously improve the digital literacy of farmers, improve their awareness of cultivated land protection, and continuously strengthen their digital use ability.

## Introduction

Ensuring food security is of paramount importance for the country safety and serves as the cornerstone for the steady economic development, social stability, and harmony of the new era. Cultivated land, being an essential component of agricultural production, is vital for maintaining food security, promoting social stability, and fostering sustainable development [1]. The No.1 Central Document for 2024 issued by the Communist Party of China and the State Council calls for a unified system to protect arable land’s quantity, quality, and ecology, enhancing governance of degraded lands, intensifying black soil protection, boosting organic matter in arable land, promoting local fallow land utilization, and prioritizing grain and economic crop production on suitable lands. However, the interplay of various factors, including the rise in extreme weather occurrences in recent years [2,3], intensifying global geopolitical tensions, the over-reliance on chemical fertilizers and pesticides by agricultural producers [4], and unsustainable farming methodologies, has exacerbated the conflict among food security, economic advancement, and the preservation of arable land. This issue has become increasingly pronounced in numerous densely populated regions of China, where land resources are limited. As the population continues to surge and urbanization progresses, the inflexibility of diminishing arable land and the ongoing deterioration of land quality are becoming increasingly apparent [5,6]. In terms of the quantity of arable land, the “Statistical Communique on China’s Natural Resources for 2023”, published by the Ministry of Natural Resources in early 2024, indicates that China’s arable land has diminished to 127.58 million hectares, resulting in a per capita arable land area of approximately 0.09 hectares, which is significantly below the global average. Concerning the quality of arable land, the “Methods for Investigation, Monitoring, and Evaluation of Arable Land Quality” and the “Grades of Arable Land Quality” (GB/T 33469-2016) categorize arable land into ten distinct grades. Current data reveals that the average national arable land quality grade stands at a mere 4.76. This information underscores the formidable and critical challenge of safeguarding national food security and enhancing farmland protection.

To fully realize the potential value of arable land, it is imperative to not only support agricultural development and ensure food security but also to evaluate the intensive effect of cultivated land and utilize resources judiciously. In this context, advocating for measures to preserve the quality of arable land is vital for protecting food security, building a robust agricultural framework, and attaining sustainable agricultural advancement. Numerous scholars have undertaken comprehensive empirical investigations into the determinants influencing farmers’ cultivated land quality protection. These factors can primarily be divided into two categories: internal factors, which encompass individual characteristics such as age and educational attainment [7], familial attributes including labor dynamics, land area, and land fragmentation [6,8], as well as cognitive aspects like household perceptions and awareness of land conservation [9,10]. The second category consists of external environmental factors, which include social services [11,12] and land tenure arrangements [13,14].

In the process of vigorous development of digital technology, digital technology empowers the green development of agriculture [15,16]. In recent years, researchers have concentrated on the impact of digital technology on agricultural production practices, encompassing internet usage [17], the adoption of digital technologies [18], and the process of digital transformation [19], among other aspects. In contrast to digital technology, digital literacy is recognized as a vital form of human capital that empowers individuals to effectively select, integrate, and apply information for the resolution of practical challenges through the use of digital technology [20,21]. Farmers with elevated levels of digital literacy demonstrate a heightened capacity to leverage digital technology, thereby enhancing their likelihood of engaging in cultivated land quality protection practices. Consequently, it is primarily those farmers with advanced digital literacy who can more effectively utilize digital technology, mitigate excessive costs associated with land quality conservation efforts, and secure income premiums from high-quality, environmentally sustainable agricultural products. However, studies related to this topic are limited. An urgent question is: Can digital literacy serve as a catalyst for promoting farmers’ land quality protection practices? If this logic holds, in what ways can digital literacy facilitate the implementation of these protection behaviors among farmers? Is there any heterogeneity present?

To fill the gaps in existing research, this study utilizes survey data collected from in Jiangxi Province and constructs O-probit models alongside mediating effect models to analyze the impact of digital literacy on farmers’ cultivated land quality protection behaviors, as well as the differences in this impact. The goal is to offer tailored recommendations for enhancing farmland conservation and safeguarding food security. Thus, we attempt to make three contributions. First, we apply the O-probit model to estimate the influence of digital literacy on farmers’ cultivated land quality protection behaviors, enriching and expanding the literature on the factors influencing cultivated land quality protection behaviors. Second, it undertakes empirical analysis utilizing micro-data to establish a robust theoretical foundation for the development and refinement of pertinent policies. Third, we further investigate the heterogeneous effects. Understanding how digital literacy heterogeneously affects farmers’ cultivated land quality protection behaviors helps policymakers design specific programs.

## Theoretical analysis

### The direct impact of digital literacy on farmers’ cultivated land quality protection behaviors

Information economics theory holds that prices incur certain costs during the search process, making information incomplete [22]. On one hand, digital literacy, as a new form of human capital, significantly reduces search costs and information asymmetry in transactions, breaking geographic barriers and disparities in economic development, highlighting the importance of understanding land quality protection behaviors, enhancing awareness of land protection, and thus promoting farmers’ cultivated land quality protection behaviors. On the other hand, digital literacy is a comprehensive ability that enhances farmers’ digital skills, aiding them in the correct, skillful, and accurate use of digital tools, devices, and resources [23,24], facilitating a better grasp of cultivated land quality protection techniques in the farming process, and thereby promoting farmers’ cultivated land quality protection behaviors. Based on this, the following hypothesis is proposed:

H1: Digital literacy can enhance farmers’ cultivated land quality protection behaviors.

### Indirect channel of digital literacy on farmers’ cultivated land quality protection behaviors

#### The mediating role of cultivated land protection cognition.

The theory of planned behavior posits that cognition serves as the foundation for behavior, with cognitive factors directly influencing the behavioral intentions and decisions of the actor [25]. The cognitive dimensions of farmers’ cultivated land quality protection behaviors are intricately linked to the information they receive and comprehend. Digital literacy has the potential to reshape our cognitive processes, allowing us to effectively and intuitively navigate a vast array of knowledge within the digital landscape [26,27], thereby enhancing farmers’ awareness of cultivated land protection and subsequently fostering their behaviors to protect cultivated land quality.

Under the background of digital economy, farmers are increasingly able to access a wealth of information on food safety and agricultural pollution through the Internet. This access allows them to comprehend the detrimental effects associated with the overuse of fertilizers and pesticides in agricultural practices, thereby recognizing the critical importance of cultivated land protection. As a result, there is a notable enhancement in their awareness and understanding of land conservation, which subsequently fosters behaviors aimed at safeguarding farmland quality [28]. Conversely, digital technologies are broadening the sales avenues available to farmers, facilitating direct interactions between agricultural producers and consumers through platforms such as Douyin and Taobao. This connection helps farmers acknowledge the growing consumer demand for green agricultural products, appreciate the intrinsic value of these products, and understand the significance of sustainable production practices. Consequently, this awareness bolsters their commitment to farmland protection and encourages the adoption of practices that ensure the quality of agricultural land [24]. In light of these observations, the following hypothesis is proposed:

H2: Digital literacy can promote farmers’ cultivated land quality protection behaviors through cultivated land protection cognition.

#### The mediating role of digital use ability.

Digital literacy encompasses to the capacity to comprehend and leverage information from a diverse digital platforms [29]. Within the agricultural domain, digital literacy can significantly empower farmers to access, evaluate, and implement information pertinent to their land, ultimately impacting their decision-making concerning the protection of cultivated land quality.

Initially, digital literacy enhances farmers’ ability to access and comprehend data related to farmland health and soil quality. By leveraging a variety of digital tools and platforms, including sensors, Geographic Information Systems (GIS), remote sensing technologies, farmers can obtain real-time insights into critical indicators of their land, thereby establishing a scientific basis foundation for the formulation of effective soil management and conservation strategies [30]. Furthermore, digital literacy strengthens farmers’ capacity in agricultural decision-making. Utilizing decision-making tools and models powered by digital technology, farmers can interpret and analyze soil test results, predict the impact of different agricultural practices on soil health, optimize resource utilization efficiency in agricultural production processes, ultimately minimizing soil quality degradation and environmental impact to the greatest extent [31]. Additionally, digital literacy fosters information exchange and collaboration among farmers, as well as between farmers and agricultural experts, government agencies. Through digital platforms, farmers can participate in communities and networks, sharing best practices and experiences from laboratory and on-site experiments [32] which not only broadens their knowledge base but also heightens their awareness and ability to take action on farmland protection issues. Lastly, the enhancement of digital literacy can effectively increase farmers’ receptiveness of emerging agricultural technologies and innovations. Technological advancements in agriculture, such as precision farming and intelligent agricultural management systems, depend on digital technology support, these technologies can boost agricultural productivity while mitigating adverse effects on the environment and cultivated land quality. Based on this, the following hypothesis is proposed:

H3: Digital literacy can promote farmers’ cultivated quality protection behavior through digital use ability.

**Fig 1:**
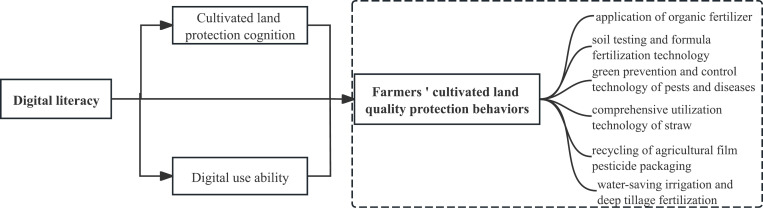
Theoretical framework diagram.

Based on the above theoretical analysis and research assumptions, this study proposes a logical framework diagram (Fig 1).

## Date and methods

### Date source

The data is derived from the survey data of “ double hundred and double thousand “ farmers in Jiangxi Province in July 2023. Through the stratified sampling method, the field survey was carried out around 11 cities, 24 counties (cities, districts), 72 townships and 216 administrative villages in Jiangxi Province. The survey covered the characteristics of farmers, industrial prosperity, ecological livability, and other pertinent topics. A total of 2160 questionnaires were generated. After eliminating invalid samples with incomplete survey information, a total of 1984 valid samples were retained. Among them, 749 farmers engaged in the protection of cultivated land quality, accounting for 37.75% of the overall sample size. Written consent was obtained from all the participants.

### Variables definition

The dependent variable is farmers’ cultivated land quality protection behaviors. The quality protection behavior of cultivated land in this study includes the application of organic fertilizer, soil testing and formula fertilization technology, green prevention and control technology of pests and diseases, comprehensive utilization technology of straw, recycling of agricultural film pesticide packaging, water-saving irrigation and deep tillage fertilization. Among the survey samples, 525 households, 128 households, 142 households, 119 households, 201 households and 86 households participated in the six kinds of farmland quality protection behaviors respectively. Among them, the largest number of participating households is the application of organic fertilizer, accounting for 70.09% of the farmers participating in the protection of cultivated land quality, the least number of households involved was water-saving irrigation and deep tillage fertilization, accounting for 11.48%.

The independent variable is digital literacy. Based on the survey data of “Double Hundred and Thousand” in Jiangxi Province, the factor analysis method is used to measure digital literacy from five dimensions: information communication literacy, content creation literacy, information and data literacy, financial use literacy, and problem solving literacy. As shown in Table 1, the KMO test value of principal component factor analysis is 0.899, which is greater than 0.7 and is suitable for principal component factor analysis.

**Table 1:** Definition of digital literacy.

Variable	Item	Index
Digital literacy	Information communication literacy	Can you pass the network information you have to others through WeChat?
		Can you participate in the communication of important public affairs in the village through WeChat and other software?
	Content creation literacy	Can you use your mobile phone to express your views and opinions online?
		Can you use mobile video software to create or publish short videos?
	Information and data literacy	Can you use the Internet to search for the agricultural information you need?
		Can you judge the authenticity of the information obtained through the network channel?
	Financial use literacy	Do you use WeChat, Alipay and other third-party payment?
		Do you use Yu ‘e Bao, online banking to buy Internet financial products or use Ant Credit Jingdong digital credit products?
	Problem solving literacy	Do you want to solve problems in production and life by querying the network?
		APP Can you download the mobile APP independently?

The mediating variables are cultivated land protection cognition and digital use ability. Cultivated land protection cognition is measured using “Is participation in land quality protection behaviors significantly improving the environment?”. Digital use ability is measured using “Are digital technologies such as the Internet, drones, and artificial intelligence utilized in production processes?”

The control variables include individual characteristics of farmers (age, gender, education level, health status, experience of party members and Village cadre experience), household farming characteristics (number of labor force, planting area, proportion of agricultural income, farmers’ cooperatives). Table 2 displays the definition and descriptive statistical analysis for each variable.

**Table 2:** The definition and descriptive statistical analysis of each variable.

Type	Variable	Description	Mean	Standard deviation
Dependent variables	Farmers’ cultivated land quality protection behaviors	The number of measures involved in the protection of the quality of cultivated land	0.861	1.079
Independent variables	Digital literacy	Calculated according to the factor analysis method	0.000	1.000
Control variables	Sex	Male = 1, female = 0	0.798	0.401
	Age	Age of farmer (years)	57.718	12.001
	Education	Below primary school = 1, primary school = 2, junior high school = 3, high school (secondary school) = 4, college and above = 5	2.912	1.006
	Health staus	Divided into 5 grades according to the degree of importance	3.822	1.029
	Party member	Whether farmer is party member (0 = no;1 = yes)	0.320	0.467
	Village cadre experience	Whether farmer is village cadre experience (0 = no;1 = yes)	0.281	0.449
	Labor	Number of persons in household (persons)	2.946	2.468
	Planting area	Logarithm of planting area(mu)	1.786	1.456
	The proportion of agricultural income	Proportion of agricultural incom to household income	0.265	0.347
	Farmers’ cooperatives	Whether farmer is a members of cooperatives (0 = no;1 = yes)	0.218	0.413
Mediating Variables	Cultivated land protection cognition	“Does participating in the protection of the quality of cultivated land have an obvious effect on improving the environment? Divided into 5 grades according to the degree of importance	3.863	0.913
	Digital use ability	Are digital technologies such as the Internet, drones and artificial intelligence utilized in production? (0 = no;1 = yes)	0.102	0.303

### Model settings

#### O-probit model.

In this paper, farmer’s cultivated land quality protection behaviors are selected as the explained variable, combined with the research methods of existing scholars [18] (Huang et al., 2022). Considering that the explained variable is an ordered categorical variable, the O-probit model is used to study the impact of digital literacy on farmer’s cultivated land quality protection behaviors. The specific model is as follows:


$$Y=\alpha_{0}+\alpha_{1}X+\alpha_{2}Control+\varepsilon$$ (1)

In equation 1, *Y* denotes the number of farm households adopting the above six farmer ‘s cultivated land quality protection behaviors; *X* denotes digital literacy; Control is control variables; $\alpha_{0},\alpha_{1},\alpha_{2}$ are the coefficients to be estimated; *ε* is the random disturbance term.


$$Y= \{\begin{array}{l} 0,ifY^{*}\leq r_{0}\\ 1,ifr_{0}<Y^{*}\leq r_{1}\\ 2,ifr_{1}<Y^{*}\leq r_{2}\\ 3,ifr_{2}<Y^{*}\leq r_{3}\\ 4,ifr_{3}<Y^{*}\leq r_{4}\\ 5,ifr_{4}<Y^{*}\leq r_{5}\\ 6,ifY^{*}>r_{5} \end{array}$$ (2)

In equation 2, $Y_{*}$ denotes hidden variables in farmers’ cultivated land quality protection Behaviors; $r_{j}(j=0-6)$ are the unknown split points of farmer ‘s cultivated land quality protection behaviors

Assumed that ε~N(0,1), then:


$$\begin{array}{l} P(Y=0|X)=\varphi (r_{0}-\alpha_{1}X-\alpha_{2}Control)\\ P(Y=1|X)=\varphi (r_{1}-\alpha_{1}X-\alpha_{2}Control)-\varphi (r_{0}-\alpha_{1}X-\alpha_{2}Control)\\ P(Y=2|X)=\varphi (r_{2}-\alpha_{1}X-\alpha_{2}Control)-\varphi (r_{1}-\alpha_{1}X-\alpha_{2}Control)\\ P(Y=3|X)=\varphi (r_{3}-\alpha_{1}X-\alpha_{2}Control)-\varphi (r_{2}-\alpha_{1}X-\alpha_{2}Control)\\ P(Y=4|X)=\varphi (r_{4}-\alpha_{1}X-\alpha_{2}Control)-\varphi (r_{3}-\alpha_{1}X-\alpha_{2}Control)\\ P(Y=5|X)=\varphi (r_{5}-\alpha_{1}X-\alpha_{2}Control)-\varphi (r_{4}-\alpha_{1}X-\alpha_{2}Control)\\ P(Y=6|X)=1-\varphi (r_{0}-\alpha_{1}X-\alpha_{2}Control) \end{array}$$ (3)

In equation 3, *φ* denotes the cumulative density function of the standard normal allocation.

### Mediating effect model

In order to explore the mediating role of cultivated land protection cognition and digital use ability between digital literacy and farmers’ cultivated land quality protection behaviors, this paper refers to the research of Shen and Wang (2024) [33] and adopts the following model:


$$M=\beta_{0}+\beta_{1}X+\mu$$ (4)

$$Y=\gamma_{0}+\gamma_{1}X+\gamma_{2}Control+\sigma$$ (5)

In equations 4 and 5, *M* denotes the mediating variable, $\beta_{0},\beta_{1},\gamma_{0},\gamma_{1},\gamma_{2}$ are the coefficients to be estimated, μ,σ are the random perturbation term.

## Result analysis

### Benchmark regression results

In Table 3, Model (1) and model (2) present the regression results of the O-probit model. The findings indicate that digital literacy significantly enhances farmers’ cultivated land quality protection behaviors at a significant level of 1%. Models (3) and (4) display the the regression outcomes of the OLS model, which further confirm the significant positive influence of digital literacy on farmers’ cultivated land quality protection behaviors, thus H1 is verified. Among the control variables, both planting area and farmers’ cooperatives demonstrate a positive effect on farmers’ cultivated land quality protection behaviors.

**Table 3:** Benchmark regression results.

Variables	Farmers’ cultivated land protection behaviors			
	(1)	(2)	(3)	(4)
Digital literacy	0.271***	0.246***	0.263***	0.236***
	(0.031)	(0.042)	(0.029)	(0.039)
Sex		0.031		−0.010
		(0.093)		(0.085)
Age		0.005		0.006
		(0.004)		(0.004)
Education		0.067		0.075*
		(0.042)		(0.039)
Health staus		0.020		0.023
		(0.035)		(0.032)
Party member		−0.091		−0.084
		(0.077)		(0.072)
Village cadre experience		0.035		0.025
		(0.075)		(0.070)
Labor		0.026		0.027
		(0.018)		(0.016)
Planting area		0.099***		0.104***
		(0.026)		(0.024)
The proportion of agricultural income		0.022		0.000
		(0.099)		(0.091)
Farmers’ cooperatives		0.136*		0.136*
		(0.074)		(0.070)
N	1984	1984	1984	1984
Pseudo R^2^/R^2^	0.0229	0.0347	0.0580	0.0832
Wald chi2/F	77.67	112.35	83.679	11.529

Note:

***,** and *indicate significane at the 1%, 5% and 10% levels, respectively, and standard errors in reported in parenthese.

Table 4 further illustrates the marginal effects of each variable in model (2) on farmers’ cultivated land quality protection behaviors. The findings indicate that the probability of farmers not adopting any kind of cultivated land quality protection behaviors measures decreases by 9.3% for every unit increase in digital literacy. Conversely, the probability of engaging in one type of cultivated land quality protection behavior rises by 2.6%, while participation in two types increases by 3.2%. Additionally, the likelihood of involvement in three types of protection measures grows by 1.8%, four types by 1.0%, five types by 0.4%, and six types by 0.3%. These results suggest that higher levels of digital literacy among farmers correlate with increased engagement in cultivated land quality protection behaviors, highlighting the significant role of digital literacy in promoting such practices.

**Table 4:** Marginal effect regression results.

Variables	Number of cultivated land protection behaviors						
**0**	**1**	**2**	**3**	**4**	**5**	**6**
Digital literacy	−0.093***	0.026***	0.032***	0.018***	0.010***	0.004***	0.003**
	(0.015)	(0.005)	(0.006)	(0.004)	(0.002)	(0.001)	(0.001)
Sex	−0.012	0.003	0.004	0.002	0.001	0.001	0.000
	(0.035)	(0.010)	(0.012)	(0.007)	(0.004)	(0.002)	(0.001)
Age	−0.002	0.000	0.001	0.000	0.000	0.000	0.000
	(0.002)	(0.000)	(0.001)	(0.000)	(0.000)	(0.000)	(0.000)
Education	−0.025	0.007	0.009	0.005	0.003	0.001	0.001
	(0.016)	(0.004)	(0.005)	(0.003)	(0.002)	(0.001)	(0.001)
Health staus	−0.008	0.002	0.003	0.001	0.001	0.000	0.000
	(0.013)	(0.004)	(0.004)	(0.003)	(0.001)	(0.001)	(0.000)
Party member	0.034	−0.010	−0.012	−0.007	−0.004	−0.002	−0.001
	(0.029)	(0.008)	(0.010)	(0.006)	(0.003)	(0.001)	(0.001)
Village cadre experience	−0.013	0.004	0.004	0.003	0.001	0.001	0.000
	(0.028)	(0.008)	(0.010)	(0.005)	(0.003)	(0.001)	(0.001)
Labor	−0.010	0.003	0.003	0.002	0.001	0.000	0.000
	(0.007)	(0.002)	(0.002)	(0.001)	(0.001)	(0.000)	(0.000)
Planting area	−0.037***	0.011***	0.013***	0.007***	0.004***	0.002**	0.001**
	(0.010)	(0.003)	(0.003)	(0.002)	(0.001)	(0.001)	(0.000)
The proportion of agricultural income	−0.008	0.002	0.003	0.002	0.001	0.000	0.000
	(0.037)	(0.011)	(0.013)	(0.007)	(0.004)	(0.002)	(0.001)
Farmers’ cooperatives	−0.051*	0.014*	0.017*	0.010*	0.006*	0.002	0.001
	(0.028)	(0.008)	(0.010)	(0.005)	(0.003)	(0.001)	(0.001)

Note:

***

**and *indicate significane at the 1%, 5% and 10% levels, respectively, and standard errors in reported in parenthese.

### Robustness tests

In order to verify the robustness of the above results, this paper uses three methods as follows: replacement of regression model, restricted subsample and replacement of dependent variable to test the robustness, as shown in Table 5.

**Table 5:** Robustness test results.

Variables	Replacement of Regression Model (5)	Restricted Subsample (6)	Replacement of Dependent Variable (7)
Digital literacy	0.404***	0.367***	0.195***
	(0.072)	(0.052)	(0.043)
Control variables	Yes	Yes	Yes
Pseudo R^2^	0.032	0.044	0.099
Wald chi2	105.03	104.87	232.58
N	1984	1984	1984

Note:

***

**and *indicate significane at the 1%, 5% and 10% levels, respectively, and standard errors in reported in parenthese.

Initially, model (5) substitutes the O-probit model with the O-logit model. The findings indicate that digital literacy positively influences farmers’ cultivated land quality protection behaviors. Subsequently, model (6) employs a limited sample approach. Given the lower cognitive levels of the aging labor force, their diminished learning capacity, and a reduced receptiveness to new concepts, the representativeness of farmers’ cultivated land quality protection behaviors is compromised. Consequently, individuals over the age of 64 are excluded from the sample. The results reveal that digital literacy continues to enhance farmers’ cultivated land quality protection behaviors. Lastly, model (7) utilizes a method of substituting the explanatory variable, replacing “cultivated land quality protection behaviors” with “at least one cultivated land quality protection behavior” and transforming the dependent variable from an ordered to a binary variable. The Probit model is employed for estimation. The results demonstrate that the findings are consistent with the significance and direction of the previous results.

### Mediating effect

Table 6 presents the mediating influence of cultivated land protection cognition and digital use ability. Both model (8) and model (9) demonstrate that the mediating effect of cultivated land protection cognition is positive and significant in the impact of digital literacy on farmers’ cultivated land quality protection behaviors. This shows that there is a transmission mechanism of “digital literacy → improving cultivated land protection cognition → cultivated land quality protection behaviors “, thereby substantiating the H2 hypothesis.. Similarly, both model (10) and model (11) illustrate that the mediating effect of digital use ability is positive and significant in the impact of digital literacy on farmers’ cultivated land quality protection behaviors. This indicates that there is a transmission mechanism of “ digital literacy → improving digital use ability → cultivated land quality protection behaviors “, which verifies H3.

**Table 6:** Mediating effect results.

Variables	Cultivated land protection cognition (8)	Farmers’ cultivated land protection behaviors (9)	Digital use ability (10)	Farmers’ cultivated land protection behaviors (11)
Digital literacy	0.124***	0.210***	0.066***	0.212***
	(0.021)	(0.040)	(0.007)	(0.039)
Cultivated land protection cognition		0.124***		
		(0.032)		
Digital use ability				0.497***
				(0.095)
Control variables	Yes	Yes	Yes	Yes
N	1984	1984	1984	1984

Note:

***

**and *indicate significane at the 1%, 5% and 10% levels, respectively, and standard errors in reported in parenthese.

### Heterogeneity analysis

#### Heterogeneity of soil fertility conditions.

As far as soil fertility conditions are concerned, the utilization rate of cultivated land with good fertility is often high, and the enthusiasm of farmers to participate in the protection of cultivated land quality is stronger. Therefore, the samples are divided into good fertility and poor fertility to further explore the heterogeneity of farmers’ cultivated land quality protection behaviors under different cultivated land conditions. The model (12) and model (13) in Table 7 are the results of different cultivated land conditions. The findings indicate that under the condition of good cultivated land fertility, digital literacy can promote farmers’ cultivated land quality protection behaviors. Conversely, in conditions of low fertility, this effect is not statistically significant. The potential reason lies in the favorable soil fertility conditions that encourage farmers to engage in practices to preserve soil quality.

**Table 7:** Heterogeneity analysis.

Variables	Good fertility (12)	Poor fertility (13)	High digital literacy (14)	Low digital literacy (15)
Digital literacy	0.300***	0.193	0.373***	0.156
	(0.064)	(0.129)	(0.091)	(0.098)
Control variables	Yes	Yes	Yes	Yes
N	1984	1984	1984	1984

Note:

***

** and *indicate significane at the 1%, 5% and 10% levels, respectively, and standard errors in reported in parenthese.

#### Heterogeneity of digital literacy.

Digital literacy helps to improve farmers’ awareness of cultivated land protection and improve their ability to use agricultural digital data, so as to have the advantages of high resource utilization efficiency and reduce transaction and search costs, thus promoting farmers ‘ tendency to adopt cultivated land quality protection behaviors. Model (14) and model (15) in Table 7 are the results of different digital literacy. The results show that under different digital literacy, high digital literacy can promote farmers’ cultivated land quality protection behaviors, while low digital literacy cannot promote farmers’ cultivated land quality protection behaviors. This indicates that only farmers with higher levels of digital literacy possess the capability to engage in cultiviated land quality protection behavior.

## Discussion

 Arable land represents the most precious agricultural asset and serves as the primary element of production. The existing research on digital literacy and farmers’ cognition of cultivated land protection mostly focuses on the impact on farmers’ entrepreneurial behavior [23], green consumption behavior [18], etc., and has not yet fully explored the impact of farmers’ cultivated land quality protection behaviors. This paper finds that digital literacy has a positive effect on farmers’ cultivated land quality protection behaviors, which is consistent with existing research results [11–14]. In alignment with the majority of studies [24], the research shows that digital literacy can promote farmers’ cultivated land quality protection behaviors by improving farmers’ cultivated land protection cognition and enhancing their digital use ability. Furthermore, this paper also examines the heterogeneous effects of cultivated land fertility status and digital literacy level on farmers’ cultivated land quality protection behaviors, and the conclusions are consistent with findings that corroborate existing literature [33]. The purpose of this paper is to provide theoretical support for improving farmers’ digital literacy, enhancing farmers’ awareness of cultivated land protection, ensuring national food security and achieving high-quality agricultural development, and strengthening and supplementing previous research results.

Nevertheless, this research presents certain limitations. Initially, the factors affecting the quality protection behavior of cultivated land are multifaceted. This article only considers the impact of digital literacy on farmers’ cultivated land quality protection behaviors and does not consider the impact of other factors. Furthermore, this paper uses one year of data in 2023 to study the impact of digital literacy on farmers’ cultivated land quality protection behaviors. Given that the process of land cultivation is intricate and extends over time, employing a longitudinal panel data analysis would yield more precise insights. In future research, more extensive data will be used to make up for the above deficiencies, explore the impact of different influencing factors on farmers’ cultivated land quality protection behaviors, analyze the dynamic changes of farmers ‘ behavior, and ultimately provide better theoretical support for policy makers.

## Conclusion and policy recommendations

Based on survey data in 2023, this paper uses O-probit model and mediating effect model to explore the impact of digital literacy on farmers’ cultivated land quality protection behaviors. The specific conclusions are as follows: (1) Digital literacy significantly positively promotes farmers ‘cultivated land quality protection behaviors. (2) Digital literacy indirectly positively affects farmers ‘cultivated land quality protection behaviors by improving cultivated land protection cognition and enhancing digital use ability. (3) The impact of digital literacy on farmers ‘cultivated land quality protection behaviors is heterogeneous. Under the conditions of good soil fertility and high digital literacy, farmers are more inclined to participate in cultivated land quality protection behaviors.

In order to give full play to the role of digital literacy on farmers’ cultivated land quality protection behaviors. The local governments should strengthen the construction of rural digital technology infrastructure. Investment in new types of infrastructure such as rural digital signals, the Internet of Things, and digital platforms should be increased to enhance the level of rural digitization, networking, and intelligence. The enhancement of digital literacy’s capacity to foster farmers’ cultivated land protection behaviors must be prioritized. It is essential to implement a diverse range of digital skills training programs, utilizing various mediums such as video, animation, and live broadcasts. Specialized strategies should be developed to enhance the pathway of digital literacy-awareness. Ecological information pertaining to the conservation of arable land is disseminated via public platforms, broadcast media, and other channels to motivate them to participate in farmers’ cultivated land quality protection behaviors.

## Supporting information

S1 Text Data. (XLSX)
